# Retrivability in The Danish National Hospital Registry of HIV and hepatitis B and C coinfection diagnoses of patients managed in HIV centers 1995–2004

**DOI:** 10.1186/1471-2288-8-25

**Published:** 2008-04-25

**Authors:** Niels Obel, Hanne Reinholdt, Lars H Omland, Frederik Engsig, Henrik T Sørensen, Ann-Brit E Hansen

**Affiliations:** 1 Department of Infectious Diseases, Copenhagen University Hospital, Rigshospitalet, Copenhagen, Denmark; 2 Research Service Unit, National Board of Health, Copenhagen, Denmark; 3 Department of Clinical Epidemiology, Aarhus University Hospital, Aarhus, Denmark; 4 Department of Epidemiology, Boston University, Boston, Massachusetts, USA; 5 Department of Infectious Diseases, Odense University Hospital, Odense, Denmark

## Abstract

**Background:**

Hospital-based discharge registries are used increasingly for longitudinal epidemiological studies of HIV. We examined completeness of registration of HIV infections and of chronic hepatitis B (HBV) and hepatitis C (HCV) coinfections in the Danish National Hospital Registry (DNHR) covering all Danish hospitals.

**Methods:**

The Danish HIV Cohort Study (DHCS) encompasses all HIV-infected patients treated in Danish HIV clinics since 1 January 1995. All 2,033 Danish patients in DHCS diagnosed with HIV-1 during the 10-year period from 1 January 1995 to 31 December 2004 were included in the current analysis. We used the DHCS as a reference to examine the completeness of HIV and of HBV and HCV coinfections recorded in DNHR. Cox regression analysis was used to estimate hazard ratios of time to diagnosis of HIV in DNHR compared to DHCS.

**Results:**

Of the 2,033 HIV patients in DHCS, a total of 2,006 (99%) were registered with HIV in DNHR. Of these, 1,888 (93%) were registered in DNHR within one year of their first positive HIV test. A CD4 < 200 cells/μl, a viral load >= 100,000 copies/ml and being diagnosed after 1 January 2000, were associated with earlier registration in DNHR, both in crude and adjusted analyses. Thirty (23%) HIV patients registered with chronic HBV (n = 129) in DHCS and 126 (48%) of HIV patients with HCV (n = 264) in DHCS were registered with these diagnoses in the DNHR. Further 17 and 8 patients were registered with HBV and HCV respectively in DNHR, but not in DHCS. The positive predictive values of being registered with HBV and HCV in DHCS were thereby estimated to 0.88 and 0.97 and in DNHR to 0.32 and 0.54.

**Conclusion:**

The study demonstrates that secondary data from national hospital databases may be reliable for identification of patients diagnosed with HIV infection. However, the predictive value of co-morbidity data may be low.

## Background

Since the first AIDS cases were reported, AIDS and HIV infection have been the subject of a wide spectrum of epidemiologic studies [1-3]. The characteristics of HIV infection make it both ideal and highly relevant for epidemiological research: valid diagnostic tools, well-described risk factors, unambiguous disease outcomes, a high impact on morbidity and mortality, worldwide dissemination, and huge economic consequences commanding the attention of national and international leaders.

During recent decades, patient registration in computerized hospital database systems has become common [1]. Although they are not designed primarily for epidemiological studies, such hospital-based registries are often used as a quick, convenient way to create huge datasets for epidemiological studies of multiple outcomes [4,5]. However their data may not always be entirely accurate or complete.

Due to shared routes of infection, many HIV-infected patients are coinfected with hepatitis B (HBV) or hepatitis C (HCV). Coinfection with these viruses, especially with HCV, is a strong prognostic marker for effectiveness of antiretroviral treatment [6,7]. Furthermore, HBV and HCV coinfection may influence the timing and choice of antiretroviral treatment. In prognostic studies of HIV, it is thus important to include HBV and HCV status, both to predict prognosis in patient subgroups [8] and to adjust for confounding [9].

The present study quantified the completeness of registration of diagnoses of HIV infection and of chronic HBV and HCV coinfections in the Danish National Hospital Registry (DNHR), based on a comparison with data collected by the Danish HIV Cohort Study (DHCS).

## Methods

### Setting

As of 1 January 2007 Denmark had a population of 5,447,084 (Statistics Denmark), with an estimated HIV prevalence of 0.07% among adults [10]. Medical care, including antiretroviral treatment, is tax-paid and provided free-of-charge to all HIV-infected residents of Denmark. Treatment of HIV infection is restricted to eight specialized medical centres, where patients are seen on an outpatient basis at intended intervals of 12 weeks. During our study's follow-up period, national criteria for initiating highly active antiretroviral treatment (HAART) were HIV-related disease, acute HIV infection, pregnancy, CD4 cell count < 300 cells/μl, and, until 2001, plasma HIV-RNA > 100,000 copies/ml.

### Study population and data collection

#### Study subjects

DHCS encompasses all HIV-infected patients treated in the above described Danish HIV clinics since 1 January 1995. Patients are identified from the lists of HIV patients in the eight medical centres, as well as from local data-sources in these centers of patients tested for HIV RNA and/or CD4 cell counts. Because HIV patients undergo tests for HIV-RNA and CD4 count in the centers at least once a year, the risk of DHCS failing to capture HIV-infected patients seen in the centres is negligible. Further the fact that antiretroviral treatment is in Denmark restricted to these eight clinics ensures that very few patients diagnosed with HIV remains unrecognised by DHCS. In Denmark treatment with antiretroviral drugs is restricted to the eight medical centers participating in DHCS. DHCS has been described elsewhere [10]. The date of HIV diagnosis, dates of AIDS-defining diseases and HBV and HCV status, are registered for each patient in DHCS as well as AIDS defining events, antiretroviral treatment, HIV-RNA, CD4 counts etc. The data in DHCS are updated on a yearly basis by research nurses or physicians. HBV and HCV status are reviewed by a trained physician or study nurse at the centres to ensure data quality.

The present study includes all 2,033 DHCS patients who met the following criteria: residence in Denmark at diagnosis (*i.e., *recorded in the Danish Civil Registration System), diagnosed with HIV after 31 January 1994 and before 1 January 2005, and age 16 years or older at time of diagnosis. Patients positive for the hepatitis-B S antigen on screening are categorised as having chronic HBV in DHCS. Patients positive for HCV serology and/or with positive HCV RNA are categorised as HCV patients.

### DNHR

Hospitalisation data for study subjects were obtained from DNHR, which was established in 1977 mainly for administrative and management purposes [11]. This registry includes primary diagnosis as well as co-morbidities [ICD-8 codes until the end of 1993, ICD-10 codes thereafter (ICD-9 has never been used in Denmark)] and procedure codes for patients treated in Danish hospitals. Diagnoses are coded by the hospital physicians at discharge for inpatients and at the initial consultation for the outpatients. Starting on 1 January 1995, the registry was expanded to include outpatients. As the current study is limited to patients diagnosed after 31 December 1994, only ICD-10 codes were relevant. The first date on which a patient was admitted to or seen as an outpatient at a Danish hospital with one of the following diagnosis codes was defined as the DNHR diagnosis date:

HIV: B20.0-B24.9

Chronic hepatitis B: B18.0 or B18.1

Chronic hepatitis C: B18.2

In a sensitivity analysis we expanded these groups to include nonspecific viral hepatitis as follows:

Chronic hepatitis B and nonspecific chronic viral hepatitis: B18.0, B18.1, B18.8, B18.9

Chronic hepatitis C and nonspecific chronic viral hepatitis: B18.2, B18.8, B18.9

### Statistical analysis

The numbers of study patients registered with HIV in DNHR and with HBV or HCV coinfection in DNHR and in DHCS were determined. We calculated the completeness (as a measure of sensitivity) of DNHR data as the number of HIV patients recorded with a diagnosis in DNHR divided by the number of patients registered with the diagnosis in DHCS (the reference) [12]. We were not able to compute the predictive value of a HIV diagnosis in the DNHR since we did not have permission from the Danish Data Protection Agency to identify HIV cases recorded only in the DNHR.

Kaplan-Meier analyses were used to construct time-to-event curves. Time was calculated as time elapsed from the DHCS HIV diagnosis date to the registration date of the first HIV diagnosis in DNHR. For patients not registered with HIV in DNHR, time was calculated from the DHCS date of HIV diagnosis to death or to 1 January 2007 (date of last observation in DNHR), whichever came first. [No one was diagnosed with HIV after death in either DNHR or DHCS]. In a few cases the patient was registered with HIV in DNHR before the diagnosis was registered in DHCS, which would give negative time in time to event analysis. In these cases for technically reasons time from registration in DHCS to registration in DNHR was defined to zero.

Cox regression analysis was used to estimate hazard ratios (HR) as a measure of the relative risk of being registered with an HIV diagnosis in DNHR. The assumption of proportional hazards was assessed graphically. Age (> 40 years vs. <= 40 years), CD4 count (>= 200 vs. < 200 cells/μl) and HIV-RNA (>= 10^5 ^vs. < 10^5 ^copies/ml) at diagnosis, gender, route of infection, centre and calendar time (diagnosed before vs. on or after 1 January 2000) were included in the analysis as categorical variables.

Patients registered in DNHR within three months of the date of their first HIV diagnosis according to DHCS were categorized as early registrations. Patients registered in DNHR more than three months after DHCS registration were considered late registrations. The mortality rate ratio (MRR) for the post three-month period was calculated for this group compared with the patients diagnosed later.

Data analysis was performed using SPSS statistical software, Version 15.0 (Norusis; SPSS Inc., Chicago, Illinois, USA). The study was approved by the Danish Data Protection Agency.

## Results

**Figure 1:**
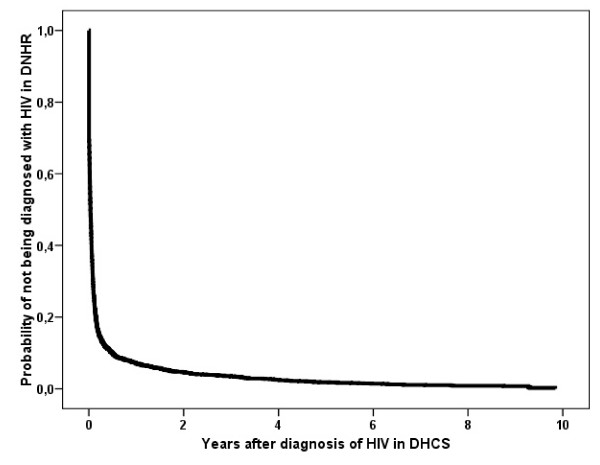
Kaplan-Meier curve of time to registration with an HIV diagnosis in the Danish National Hospital Registry from the date of first HIV positive test, according to the Danish HIV Cohort Study.

Our study included the 2,033 Danish patients diagnosed with HIV-1 according to DHCS during the 10-year period from 1 January 1995 to 31 December 2004. Baseline characteristics are shown in Table 1. Of these, a total of 2,006 (99%) were registered in DNHR with HIV (Table 2) and 1,888 (93%) were registered in DNHR within one year of the first positive HIV-1 test (Figure 1). Twenty-four (1.2%) patients were diagnosed with HIV in DNHR more than one year before registration in DHCS. In the DHCS population, 96% of patients were seen in one of the eight HIV treatment centres within one year of HIV diagnosis.

**Table 1:** Characteristics of HIV patients registered in the Danish HIV Cohort Study.

Total number of patients	2,033
Male (%)	1,496 (73.6)
Age (years, median, IQR)	36.4 (30.1 – 44.8)
Race	
Caucasian (%)	1,530 (75.3)
Black (%)	331 (16.3)
Asian (%)	111 (5.5)
Other and unknown (%)	61 (3.0)
Route of infection	
Homosexual (%)	822 (40.4)
Heterosexual (%)	908 (44.7)
Intravenous drug use (%)	180 (8.9)
Other/unknown (%)	123 (6.1)
CD4 count/ul at diagnosis (median, IQR)	290 (110 – 490)
Log (HIV-RNA/ml) at diagnosis (median, IQR)	4.71 (3.97 – 5.30)

**Table 2:** Registrations of HIV, chronic hepatitis B, and chronic hepatitis C infections in the Danish National Hospital Registry (DNHR) for 2,033 HIV patients identified in the Danish HIV Cohort Study (DHCS).

	Registered with the disease in DHCS and DNHR	Registered with the disease in DHCS but not in DNHR	Registered with the disease in DNHR but not in DHCS	Not registered with the disease in either DHCS or DNHR
HIV	2,006 (99%)	27 (1%)	Not applicable	Not applicable
Chronic hepatitis B (B18.0, B18.1)*	30 (23%)	99 (77%)	17	1,887
Chronic hepatitis B including B18.8 and B18.9*	42 (33%)	87 (67%)	30	1,874
Chronic hepatitis C (B18.2)*	126 (48%)	138 (52%)	8	1,761
Chronic hepatitis C including B18.8 and B18.9*	129 (49%)	135 (51%)	28	1,741

*Refers to the ICD-10 codes used to identify patients in DNHR.

The percentage is based on the total number of patients registered in DHCS.

### Predictors of registration of HIV diagnosis in DNHR

Being diagnosed after 1 January 2000 or with a baseline CD4 < 200 cells/μl or HIV-RNA >= 100,000 copies/ml were associated with increased change of registration of HIV diagnosis in DNHR, both in crude and adjusted analyses (Table 3). In the adjusted analysis, female gender was associated with an HIV diagnosis appearing earlier in DNHR. Route of infection, age, race and treatment centre did not have a major impact on the likelihood of being registered in DNHR.

**Table 3:** Relative risk (hazard ratio [HR]) of being registered with HIV in the Danish National Hospital Registry according to characteristics and treatment centre.

	Crude HR (95% CI)	Adjusted HR (95% CI)
Male gender (reference)	1	1
Female gender	1.05 (0.95–1.16)	1.15 (1.02–1.30)
Caucasian (reference)	1	1
Non-Caucasian	1.02 (0.92–1.13)	0.93 (0.82–1.05)
Age > 40 years (vs. <= 40 years)	1.12 (1.02–1.22)	1.07 (0.97–1.18)
CD4 count < 200 cells/μl (vs. ≥ 200 cell/μl)	1.45 (1.32–1.59)	1.38 (1.25–1.52)
Viral load > 100,000 copies/ml (vs. ≤ 100,000 copies/ml)	1.32 (1.21–1.45)	1.14 (1.03–1.26)
Heterosexual (reference)	1	1
Homosexual	1.01 (0.92–1.11)	1.04 (0.93–1.17)
Intravenous drug use	0.85 (0.72–1.00)	0.87 (0.73–1.04)
Other/unknown	1.05 (0.86–1.27)	1.07 (0.88–1.31)
Diagnosed before year 2000 (reference)	1	1
Diagnosed after 1 January 2000	1.40 (1.28–1.53)	1.40 (1.28–1.53)
**Centre**		
Center 1	1	1
Center 2	1.03 (0.93–1.14)	1.06 (0.95–1.17)
Center 3	1.08 (0.91–1.27)	1.12 (0.94–1.32)
Center 4	0.87 (0.75–1.02)	0.91 (0.78–1.07)
Center 5–8*	0.99 (0.86–1.15)	0.98 (0.83–1.14)

* The four smallest centers in the Danish HIV Cohort Study (DHCS)

### Registration and mortality

**Figure 2:**
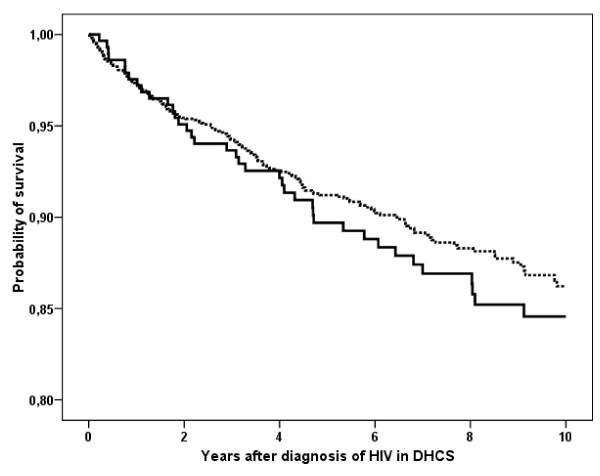
Kaplan-Meier curve of the time to death for patients with diagnoses registered early in the Danish National Hospital Registry [within 3 months of HIV diagnosis in the Danish HIV Cohort Study] [broken line] and for patients diagnosed later (after 3 months) [solid line].

To investigate the potential association between early registration in DNHR and mortality, we compared mortality among patients with a diagnosis in DNHR within 3 months of a diagnosis in DHCS with mortality among those diagnosed later. There was no statistically significant difference in risk of death in the group registered early vs. the group registered late (Figure 2, MRR = 0.87: 95% CI, 0.61 – 1.24).

### Hepatitis B and C

Details of HBV and HCV registration in DNHR and DHCS are provided in Table 2. Only 30 (23%) HIV patients documented to have chronic HBV in DHCS (n = 129), and 126 (48%) HIV patients documented to have HCV in DHCS (n = 264) were registered with these diagnoses in DNHR. Of note, 17 patients were registered with HBV (ICD10: B18.0 and B18.1) and 8 with HCV (ICD10: B18.2) in DNHR who did not have a corresponding record in DHCS (table 2). Assuming that the correct numbers of HBV and HCV are 129 + 17 = 146 and 264 + 8 = 272, the positive predictive values of being registered with HBV and HCV in DHCS were 0.88 and 0.97 and in DNHR 0.32 and 0.54 respectively.

In a sensitivity analysis, we categorized patients registered with HBV or nonspecific hepatitis as diagnosed with HBV and patients registered with HCV or nonspecific hepatitis as diagnosed with HCV. However, even when these diagnoses were combined, HBV and HCV remained incompletely documented in DNHR compared to DHCS (Table 2).

The registration in DNHR of chronic hepatitis infections for the 2,033 HIV patients in DHCS increased over time. 26% and 42% of all DHCS-established HBV and HCV coinfections were registered in DNHR in the 1995–1999 period, versus 41% (p = 0.05) and 57% (p = 0.01), respectively, for the 2000–2004 period.

## Discussion

We found almost complete registration of HIV diagnoses in DHNR. In contrast, co-infection with HBC and HCV were poorly captured.

A unique opportunity to compare two independent, nationwide registries of HIV infection allowed this study to be undertaken. The population-based DHCS was used as a reference to validate the number of HIV diagnoses in DNHR. We documented that DNHR effectively captures the diagnosis of HIV in all patients with at least one contact with an HIV hospital treatment centre. The level of completeness was close to 100%, with more than 90% of patients registered in DNHR within a year of their first HIV-positive test being documented in DHCS.

One study limitation is the unknown number of persons diagnosed with HIV who have not sought hospital care. Thus, while we can conclude that HIV patients with a hospital contact are captured by DNHR, we cannot use DNHR to determine the total number of Danish patients diagnosed with HIV. However, as nearly 95% of the patients in DHCS are seen in an HIV treatment centre within a year of HIV diagnosis, we assume that this bias is small. Twenty-four patients had a HIV diagnosis captured in the DNHR more than one year before registration in DHCS. This is not surprising, as clinical experience shows that not all patients disclose a previous HIV diagnosis when they present to an HIV treatment centre. Also some of these patients may be misclassified in DHCS.

As mentioned, we did not have permission to identify HIV cases recorded only in DNHR, and thus it was not within the aim of this study to validate the predictive value of an HIV-diagnosis recorded in DNHR. We consider this an acceptable limitation as the positive predicted value is expected to be high. HIV is unambiguously diagnosed by presence of specific antibodies detected by well-validated assay and reports of patients falsely diagnosed with HIV in Danish Hospitals are extremely rare.

Registration of HIV in DNHR was influenced slightly by demographic and HIV-associated factors. Thus, patients with signs of more advanced HIV disease (higher viral load and lower CD4 count at presentation), and patients diagnosed in later years were more likely to be registered in DNHR. Because very few HIV patients are not registered in DNHR, this bias should have little influence on estimates of disease severity based on data from DNHR. Of not, there was no significant differences in mortality in patients registered late compared with patients registered early in DNHR indicating that the probability of being registered in DNHR is not associated with risk of subsequent death.

The completeness of HIV registration is comparable to the completeness of registration in hospital databases of acute myocardial infarction, severe bacterial infections, cancer, and major surgical procedures [13-16]. These conditions are all rather easily and unambiguously diagnosed and have important treatment indications.

In contrast to HIV, HBV and HCV were incompletely registered in DNHR and registration trends varied greatly over time. Thus, DNHR is a poor tool for determining both absolute and relative risks of HBV and HCV among HIV-positive persons. There are many potential explanations for this finding. Chronic HBV and HCV are often diagnosed after routine screening, are mainly asymptomatic and do not always require treatment [17,18]. Thus HBV and HCV infection may not be an important focus of personnel coding discharge diagnoses. The importance of completeness of data on HCV infection is exemplified in a French study that clearly demonstrated that missing diagnosis of HCV (in this study by lack of HIV serology) introduced considerable bias into a cohort study of HIV and HCV coinfection [19].

Our study makes clear that while DNHR generally has good coverage, the completeness of some specific diagnoses may be insufficient for valid epidemiological research. As important examples, HBV and HCV infections are typically diagnosed secondary to the primary HIV diagnosis and are often missing from DNHR.

This validation study was conducted in a Danish setting. Given the nature of HIV infection, its results may be generalised to other countries with similarly organised health care systems such as other Scandinavian countries.

## Conclusion

In conclusion, our study confirms the importance of considering the quality of available data and the circumstances under which they are collected before conducting registry-based studies. While HIV infection can be accurately identified using secondary data in registry-based studies, medical records or other data sources may be needed to identify specific comorbidities.

## Competing interests

Niels Obel has received research funding from Roche, Bristol-Myers Squibb, Merck Sharp & Dohme, GlaxoSmithKline, Abbott, Boehringer Ingelheim, Janssen-Cilag and Swedish Orphan. Ann Brit Eg Hansen has received honoraria from GlaxoSmithKline. Hanne Reinholdt, Lars Omland, Frederik Engsig, and Henrik Toft Sørensen have no conflicts of interest.

## Authors' contributions

Conception and design: NO. Acquisition of data: NO, HR, LHO, FE, ABH. Analysis and/or interpretation of data: NO, HTS, ABH. Drafting of the manuscript: NO and ABH. Revising the manuscript critically for important intellectual content: HR, LHO, FE, HTS. All authors approved the final version.

## Pre-publication history

The pre-publication history for this paper can be accessed here:


